# Comparative Pharmacokinetics and Local Tolerance of Tenofovir Alafenamide (TAF) From Subcutaneous Implant in Rabbits, Dogs, and Macaques

**DOI:** 10.3389/fphar.2022.923954

**Published:** 2022-07-19

**Authors:** G. J. Gatto, A. Krovi, L. Li, I. Massud, A. Holder, J. Gary, P. Mills, J. Mitchell, E. Luecke, Z. R. Demkovich, W. Heneine, J. G. García-Lerma, M. A. Marzinke, R. M. Brand, C. W. Dobard, L. M. Johnson, A. Van Der Straten

**Affiliations:** ^1^ RTI International, Research Triangle Park, NC, United States; ^2^ Division of HIV/AIDS Prevention, National Center for HIV/AIDS, Viral Hepatitis, STD and TB Prevention, Centers for Disease Control and Prevention, Atlanta, GA, United States; ^3^ Neuropathology, StageBio, Frederick, MD, United States; ^4^ Department of Comparative Medicine, Tulane University, New Orleans, LA, United States; ^5^ Department of Pathology, Johns Hopkins University School of Medicine, Baltimore, MD, United States; ^6^ Department of Medicine, Johns Hopkins University School of Medicine, Baltimore, MD, United States; ^7^ Department of Internal Medicine, University of Pittsburgh School of Medicine, Pittsburgh, PA, United States; ^8^ Magee-Womens Research Institute, University of Pittsburgh, Pittsburgh, PA, United States; ^9^ Department of Medicine, Center for AIDS Prevention Studies (CAPS), University of California, San Francisco, CA, United States; ^10^ ASTRA Consulting, Kensington, CA, United States

**Keywords:** HIV pre-exposure prophylaxis, tenofovir alafenamide, biodegradable polymer, reservoir implants, comparative pharmacokinetics

## Abstract

The administration of antiretrovirals (ARVs) for HIV pre-exposure prophylaxis (PrEP) is highly efficacious and may benefit from new long-acting (LA) drug delivery approaches. This paper describes a subcutaneous, reservoir-style implant for the LA delivery of tenofovir alafenamide (TAF) and documents the preclinical assessment of implant safety and pharmacokinetics (PK) in New Zealand White (NZW) rabbits (3 groups of *n* = 5), beagle dogs (2 groups of *n* = 6), and rhesus macaques (2 groups of *n* = 3). Placebo implants were placed in rabbits (*n* = 10) and dogs (*n* = 12). Implant parameters, including selection of the TAF form, choice of excipient, and PCL formulation were tuned to achieve targeted concentrations of the active anabolite of TAF, tenofovir diphosphate (TFV-DP), within peripheral blood mononuclear cells (PBMCs) and mucosal tissues relevant to HIV transmission. Sustained concentrations of TFV-DP in PBMCs over 100 fmol/10^6^ cells were achieved in all animal species indicating that the implants effectively delivered TAF for 3–6 months. Unlike placebo implants without TAF, all active implants resulted in local adverse events (AEs) proximal to the implant ranging in severity from mild to moderate and included dermal inflammation and necrosis across all species. Despite these AEs, the implant performed as designed and achieved a constant drug release profile, supporting the continued development of this drug delivery platform.

## 1 Introduction

Oral administration of HIV pre-exposure prophylaxis (PrEP) is highly efficacious in preventing infection when adhering to the dosing regimen (Baeten et al., 2012; Thigpen et al., 2012). However, challenges associated with adherence to a daily oral pill for HIV PrEP have resulted in varied outcomes of clinical trials, (Osterberg and Blaschke, 2005; Dimitrov et al., 2016), highlighting the need for alternative, long-acting (LA) drug delivery approaches. The LA and continuous administration of antiretrovirals (ARVs) may improve pharmacological profiles, reduce side-effects, increase therapeutic adherence, and provide enhanced privacy and protection to the end-user. Several LA HIV PrEP products are currently under development or under consideration by the U.S. FDA including vaginal rings, (Singer et al., 2012; Ouattara et al., 2014; Nel et al., 2016; Su et al., 2017; Thurman et al., 2018; Keller et al., 2019; Baeten et al., 2021), nanosuspensions for injection, (Baert et al., 2009; Trezza et al., 2015; Penrose et al., 2016; Rogovoy BK et al., 2017; Harris et al., 2019; Begley et al., 2020; Yakubova et al., 2020; Segal-Maurer et al., 2022), microarray patches, (Ramadan et al., 2016; Boopathy et al., 2017; Mc Crudden et al., 2018; Tekko et al., 2019), and subdermal implants (Gunawardana et al., 2015; Chua et al., 2018; Pons-Faudoa et al., 2019; Gunawardana et al., 2020; Simpson et al., 2020; Su et al., 2020; Karunakaran et al., 2021; Matthews et al., 2021). A once monthly injection of cabotegravir (CAB) and rilpivirine, Cabenuva, is FDA approved for HIV treatment in individuals who are virologically suppressed (FDA, 2021). A LA injectable of CAB, Apretude, has been recently approved by the FDA for HIV PrEP in men and women (Delany-Moretlwe et al., 2021; Landovitz et al., 2021; Rocca et al., 2021). Other promising LA products have entered the pipeline of clinical trials, including an implant for the subcutaneous delivery of the highly potent nucleoside reverse transcriptase translocation inhibitor (NRTTI), islatravir (Matthews et al., 2021) and a first-in-class long-acting capsid inhibitor, lenacapavir, delivered as a 6 months injectable (Harris et al., 2019; Segal-Maurer et al., 2022).

The high efficacy and safety profile of orally administered tenofovir alafenamide (TAF), a nucleoside reverse transcriptase inhibitor (NRTI) in combination with emtricitabine (Descovy) is FDA-approved for HIV PrEP in cisgender men and transgender women, (FDA, 2019; Keller et al., 2019), and has also encouraged the development of LA delivery options for this drug (Romano et al., 2021). For example, investigators have developed implantable systems to deliver TAF subcutaneously, including refillable (Chua et al., 2018; Simpson et al., 2020) and non-biodegradable implants (Gunawardana et al., 2015; Gunawardana et al., 2020; Su et al., 2020; Karunakaran et al., 2021). One promising example includes a silicone-based implant for LA delivery of TAF, which has advanced to Phase I/II clinical trials (PACTR201809520959443) (Massud, 2021). These advancements are encouraging, but recent preclinical evidence also suggests questionable tolerability of TAF when administered subcutaneously over sustained time periods via certain drug delivery platforms (Gunawardana et al., 2020; Simpson et al., 2020; Su et al., 2020; Pons-Faudoa et al., 2021; Romano et al., 2021).

We are developing a biodegradable implant for delivery of ARVs for HIV PrEP with promising *in vitro* and *in vivo* results previously reported (Johnson et al., 2019; Li et al., 2020; Li et al., 2021a; Li et al., 2021b). This reservoir-style implant comprises poly (ε-caprolactone) (PCL), which is designed to degrade after multiple years to enable LA delivery of ARVs and could potentially eliminate the need for a medical procedure for implant retrieval. Previously, we demonstrated our PCL implant provides controlled drug release that is tunable via modifications to the surface area, the thickness of the reservoir-implant wall, and properties of the polymer (Johnson et al., 2019; Li et al., 2021b). This manuscript describes the developmental path and accompanying preclinical studies of this reservoir-style implant that is designed to release TAF. By describing various implant specifications, including drug formulations, the composition of PCL, and architectural design, we show how key attributes affect the *in vivo* performance within several animal species: NZW rabbit, beagle dog, and rhesus macaque.

## 2 Materials and Methods

### 2.1 Materials

Tenofovir alafenamide (TAF) hemifumarate (CAS No. 1392275-56-7) and TAF free base (CAS No. 379270-37-8) were sourced from BOC Sciences (Shirley, NY, United States) and are referred to as TAF salt and TAF base throughout the paper. In the rabbit and dog studies, research-grade PCL pellets from Sigma Aldrich referred to as S80 (weight average molecular weight (Mw) = 103 kDa, PD of 1.6, Cat# 440,744, St. Louis, MO, United States) were used. Medical-grade PCL referred to as PC-17 (Mw = 106 kDa, PURASORB PC-17, PD of 2.6) was obtained from Corbion (Amsterdam, Netherlands). Super refined castor oil (Batch No. 0001211513 and 0001472789) and sesame oil (Batch No. 0001162168) were obtained from Croda International (Snaith, United Kingdom) and are referred to as CO and SO throughout the paper. The commercial disposable trocar kits were obtained either from Shinva Ande Healthcare Apparatus Co., Ltd. (Zibo City, China) or Trocarkit (www.trocarkit.com).

### 2.2 Design of TAF Implants

The release rates of TAF were tailored via choice of wall thickness, PCL type, and excipient (Johnson et al., 2019). For the rabbit studies, a wall thickness of either 200 µm (Implant A) or 100 µm (Implant B) were used to control the release kinetics. Implant A and B used the CO excipient and the formulation comprised 3:1 TAF salt:CO, which showed a membrane-controlled mechanism (Li et al., 2018).

Advancements made during the time of the rabbit studies showed an improved stability of TAF base in the implant, as compared to TAF salt (Yakubova et al., 2020). Therefore, all subsequent *in vivo* studies (Implant C and Implant D) utilized TAF base in the drug formulation. Furthermore, PC-17 was used to fabricate implants for the studies with beagle dogs and rhesus macaques due to the higher drug release rates, as compared to S80 (Li et al., 2021a). A drug formulation of 2:1 TAF base to SO was used for the dog and macaque studies based on the targeted release rates for TAF (Li et al., 2021a). For ease of review, the different TAF implant formulations examined in this manuscript are presented in Table 1.

**TABLE 1:** Specifications of implants used in the *in vivo* studies.

Species	Implant ID	PCL	TAF	Excipient	Wall Thickness (µm)	TAF to Excipient Mass Ratio	*In vitro* Release Rate (mg/day)	TAF Mass loaded in Implant (mg)±SD	No. of Implants per animal
NZW Rabbit	Implant A	Sigma (S_80_)	TAF salt	Castor Oil (CO)	200	3:1	0.19 ± 0.07	116.4 ± 1.4	1 (left side)
	Implant Placebo A	Sigma (S_80_)	---	Castor Oil (CO)	200	---	---	---	1 (right side)
	Implant B	Sigma (S_80_)	TAF salt	Castor Oil (CO)	100	3:1	0.34 ± 0.07	135.2 ± 7.7	1 (left side) or 2 (left & right sides)
	Implant Placebo B	Sigma (S_80_)	---	Castor Oil (CO)	100	---	---	---	1 (right side)
Beagle Dog	Implant C	Sigma (S_80_)	TAF base	Castor Oil (CO)	100	3:1	0.17 ± 0.06	115.4 ± 3.3	1 (left side)
	Implant Placebo C	Sigma (S_80_)	---	Castor Oil (CO)	100	---	---	---	1 (right side)
Beagle Dog & Rhesus Macaque	Implant D	PC17	TAF base	Sesame Oil (SO)	100	2:1	0.48 ± 0.17^*^	134.9 ± 0.8	1 (left side) or 2 (left & right sides)
	Implant Placebo D	PC17	---	Sesame Oil (SO)	100	---	---	---	1 (right side)

^*^ Release rate of Implant D shown for *in vitro* studies that parallel the dog study.

### 2.3 Fabrication of TAF Implants

PCL tubes were fabricated via a hot-melt, single screw extrusion process using solid PCL pellets at GenX Medical (Chattanooga, TN, United States). All tubes were 2.5 mm in outer diameter (OD) with a wall thickness of 100 µm (Implant B, Implant C, and Implant D) or 200 µm (Implant A), as measured with a 3-axis laser measurement system and light microscopy at GenX Medical. Implants were fabricated as previously reported (Johnson et al., 2019; Li et al., 2020; Li et al., 2021a). PCL tubes were first sealed at one end using injection sealing. For the injection sealing, the PCL tube was marked and trimmed to the correct length to ultimately produce an implant with a 46 mm length that contained the drug formulation and with an additional headspace of 3 mm at each end for sealing. The initial seal was then created at one end of the implant by placing the tube over a stainless-steel rod that occupied all the tube except for a 3 mm headspace at one end, positioning a Teflon collar around the headspace to support the tube wall and injecting molten PCL into the cavity of the headspace. After the injected PCL solidified, excess PCL was trimmed, and the collar was removed to form a cylindrical seal that is compatible with commercial contraceptive trocars.

Implants A and B were filled with a mixture of TAF salt and CO at a 3:1 mass ratio, Implant C was filled with a mixture of TAF base and CO at 3:1 mass ratio, and Implant D was filled with a mixture of TAF base and SO at a 2:1 mass ratio (Table 1) each drug/excipient mixture was weighed and ground with a mortar and pestle into a smooth paste. The paste was packed into the PCL tubes that were presealed at one end using a stainless-steel spatula. The tube was filled to the 40-mm mark and the end was cleaned with a metal rod and Q-tip to remove excess drug formulation and sealed in a similar manner to the first seal. After fabrication, all implants were weighed to determine the total payload and photographed with a ruler to record the final dimensions. Paste area was measured with ImageJ (version 1.52n, National Institutes of Health) and release rates were normalized to the surface area of a full-sized implant (314 mm^2^ for an implant with OD of 2.5 mm, 40 mm in length). Both ends of the implant were not included in calculations of the implant surface area. Fabrication of placebo implants followed a similar protocol (as described above). The tubes with one sealed end were filled with the corresponding neat excipient and heat-sealed on the other end to yield placebo implants.

Prior to implantation, all implants were gamma irradiated at 18–40 kGy (Steris, Libertyville, IL, United States) and remained in the sterilization package until the implantation date.

### 2.4 *In vitro* Drug Release Studies

*In-vitro* drug release assays involved incubation of the implants in 40 ml of 1X phosphate buffered saline (PBS, pH 7.4 at 37°C) on an orbital shaker at 100 rpm. TAF species in the release media were measured by ultraviolet-visible (UV) spectroscopy at 260 nm using the Synergy MX multi-mode plate reader (BioTek Instruments, Inc., Winooski, VT, United States). The release buffer was sampled twice a week during which time the implants were transferred to 40 ml of fresh buffer to maintain sink conditions. The quantity of TAF species (TAF and tenofovir-containing species) released in the PBS buffer during the time interval was calculated, and the cumulative mass of drug release as a function of time was determined.

### 2.5 Drug Content Analysis

The purity of TAF inside the implant reservoir was evaluated by opening the implant, extracting the entire reservoir contents into an organic solution, and measuring TAF chromatographic purity using ultra-performance liquid chromatography (UPLC) coupled with UV spectroscopy (UPLC/UV). The analysis was performed using a Waters BEH C18 column (2.1 mm × 50 mm, 1.7 μm) under gradient, reversed phase conditions with detection at 260 nm. For each implant, one single aliquot was prepared and quantified by linear regression analysis against a five-point calibration curve. TAF purity was calculated as the percent of the peak area associated with TAF relative to the total peak area of TAF-related degradation products (detected above the limit of detection (LOD) ≥ 0.05%). The quantity and purity of TAF within the implant were analyzed following the *in vitro* studies which were conducted for the same duration and in parallel to the corresponding *in vivo* study in rabbits (63 days), dogs (182 days), and rhesus macaques (up to 120 days).

### 2.6 *In vivo* Studies

All animal studies were conducted in accordance with a protocol approved by the local Institutional Animal Care and Use Committee (IACUC) at Magee Womens Research Institute, Charles River Laboratories, or Centers for Disease Control and Prevention (CDC) according to the provisions of the Animal Welfare ACT, PHS Animal Welfare Policy, and the principles of the NIH Guide for the Care and Use of Laboratory Animals. The incorporation of the contralateral implantation approach allowed the assessment of localized skin irritation by comparing the active implant with its matched placebo. The contralateral implantation of the active implant with the matched placebo was used in the rabbit (Implant A and Implant B), dog (Implant C and Implant D), and rhesus macaque (Implant D) studies. The contralateral implantation of two active implants was used in the rabbit (Implant B [2x]) and in the rhesus macaque (Implant D [2x]) studies.

Rabbit: Female NZW rabbits (2–3 kg) were anesthetized with isoflurane and given an intravenous administration of glycopyrrolate. Following a small incision in the dorsal scapular region, the implants were positioned in the subcutaneous space using a 4.5 mm trocar kit. The incision was closed with sterile skin glue. Animals were monitored until they recovered. The treatment groups consisted of Implant A designed to release 0.15 mg/day (0.06 mg/kg) of TAF salt (*n* = 5), Implant B designed to release 0.42 mg/day (0.15 mg/kg) of TAF salt (*n* = 5), and two implants of Implant B designed to release 0.42 mg/day for a total of 0.84 mg/day (0.34 mg/kg) (*n* = 5) of TAF salt. Animals in group A and B also received one placebo implant (*n* = 10). The design of this study involved weekly blood sampling for the determination of plasma TAF, plasma TFV and PBMC TFV-DP concentrations. On study termination day (day 35 or day 63), target tissue (vaginal, cervical, and rectal tissues) for the determination of TFV and TFV-DP were harvested, skin biopsy near the implantation site for histological assessment were collected and implants were recovered. This study included a mid-point assessment where implants were recovered from 2 animals from each treatment group.

Dog: Female beagle dogs (10–12.5 kg) were anesthetized with isoflurane and given an intravenous administration of glycopyrrolate. Following a small incision in the dorsal scapular region, the implants were positioned in the subcutaneous space using a 2.7 mm trocar kit. The incision was closed with absorbable monofilament sutures. Animals were monitored until they recovered. The treatment groups consisted of Implant C designed to release 0.16 mg/day (0.02 mg/kg) of TAF base (*n* = 6) and Implant D designed to release 0.48 (0.04 mg/kg) of TAF base (*n* = 6). Each animal in group C and D also received one placebo implant (*n* = 12). The design of this study involved weekly blood sampling for the determination of plasma TAF, plasma TFV and PBMC TFV-DP concentrations. On study termination day (day 90 or day 180), skin biopsy near the implantation site for histological assessment were collected and implants were recovered. Following the recovery of the implants, all animals were returned to the animal stock. Like the rabbit study, the study incorporated a mid-point assessment where implants were removed from 3 animals from each treatment group.

Rhesus Macaques: Female rhesus macaques (5–8 kg) were sedated with ketamine-HCl (100 mg/ml) prior to the subcutaneous insertion of two implants (one active implant inserted dorsally in the left arm and one matched placebo implant inserted dorsally in the right arm or one active implant inserted into both arms) using a 2.7 mm trocar kit. Animals were monitored until they recovered. The treatment groups consisted of Implant D designed to release 0.35 mg/day (0.05 mg/kg) of TAF base (*n* = 3), and two implants of Implant D (one in each arm) designed to release a total of 0.7 mg/day (0.11 mg/kg) of TAF base (*n* = 3). The design of this study involved weekly blood sampling for the determination of plasma TAF, plasma TFV and PBMC TFV-DP concentrations. On study termination day (day 84 or day 140), skin biopsy near the implantation site for histological assessment were collected and implants were recovered. Following the recovery of the implants, all animals were returned to the animal stock.

### 2.7 Blood and Tissue Collection

At indicated times points, blood samples were collected and processed to plasma or peripheral blood mononuclear cells (PBMCs) to determine TAF, tenofovir (TFV), and TFV-DP. In rabbits, target tissues (vaginal, cervical, and rectal) and tissues surrounding the implant were harvested and placed in snapped frozen cryovials to determine TFV-DP.

### 2.8 Bioanalytical

Plasma concentrations of TAF and TFV and tissue concentrations of TFV were measured via previously described liquid chromatographic-tandem mass spectrometric (LC-MS/MS) methods by the Clinical Pharmacology Analytical Laboratory (CPAL) at the Johns Hopkins University School of Medicine (Hendrix et al., 2016; Hummert et al., 2018). The lower limits of quantification (LLOQ) for plasma TAF and TFV were 0.03 ng/ml and 1 ng/ml, respectively. The LLOQ for TFV in tissue was 0.05 ng/sample. Results were normalized to biopsy weights and reported as ng/mg.

TFV-DP measurements in PBMCs and tissue were determined via LC-MS/MS by the CPAL; the LLOQ for TFV-DP in PBMC and tissue was 5 fmol/samples, as previously described (Cerrone et al., 2019). Results were normalized and converted to fmol/10^6^ cells based on the lysate-specific number of PBMCs present in the sample; anabolite concentrations in tissue were normalized to biopsy weights and reported as ng/mg.

### 2.9 Pharmacokinetic Analysis

Actual time points were used in the PK analysis of TAF and TFV in plasma and PBMCs. The PK calculations were based on non-rounded median total plasma concentration-time data, calculated from pooled individual plasma concentrations at each sampling time point. The maximum serum concentration, C_max_, and time of maximum concentration, t_max_, were direct observations from the mean plasma concentration vs. time curve. Exposure of animals to TAF expressed as area under the plasma concentration-time curve from zero to the last measurable sample, AUC_(0-t)_, was obtained by means of non-compartmental analysis in PKSolver (Zhang et al., 2010). AUC_(0-t)_ was calculated by means of the linear up-log down method. For the tissue, sampling times were presented for tissue concentrations of TFV and TFV-DP.

### 2.10 Histopathology Evaluation

Tissues and skin biopsies were harvested at different time points (either at the midpoint or the completion of the study) from the surrounding implant sites and were fixed in 10% formalin and stored in 70% ethanol until assessment. The tissues were embedded in paraffin, cut into sections, and stained with hematoxylin and eosin (H&E). Histological evaluation was conducted by a blinded pathologist. A modified Draize skin irritation protocol was performed to measure local skin reactions (erythema and inflammation) to the TAF implant in the rhesus macaques.

### 2.11 Statistical Analysis

Plasma concentrations are graphically illustrated using SigmaPlot 13 (Systat Software, Inc., San Jose, CA). In the graphical visuals, plasma concentrations below the LLOQ were treated as missing and excluded from calculations. The descriptive statistical calculations were performed in SigmaPlot 13. For graphical representation and interpretation of pharmacokinetics, data are presented as median ± the standard deviation.

## 3 Results

**FIGURE 1:**
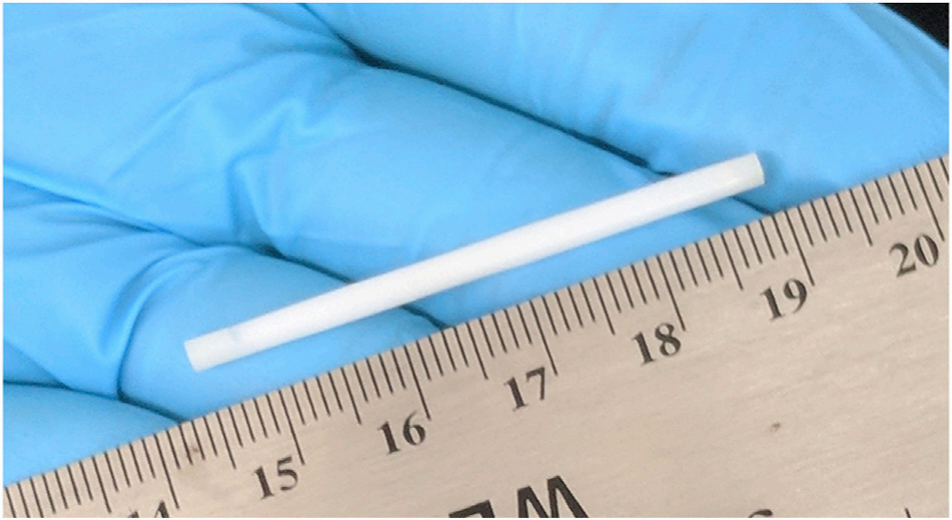
A digital camera image of the biodegradable implant. All implants were 2.5 mm in outer diameter (OD) and 46 mm in total implant length with a 40 mm reservoir and two 3 mm PCL end seals.

We designed a PCL biodegradable reservoir implant to sustainably deliver TAF (Figure 1) and assessed its drug release profile and PK properties using *in vitro* and *in vivo* assays. Besides the evaluation of TAF delivery over 3–6 months, we also assessed the safety and tolerability of the implant in multiple animal species. The results are discussed below with the focus of the feasibility and the limitations of developing a LA TAF implant for HIV PrEP.

### 3.1 *In vitro* Studies

#### 3.1.1 *In vitro* Studies Performed in Parallel to the NZW Rabbit Study

**FIGURE 2:**
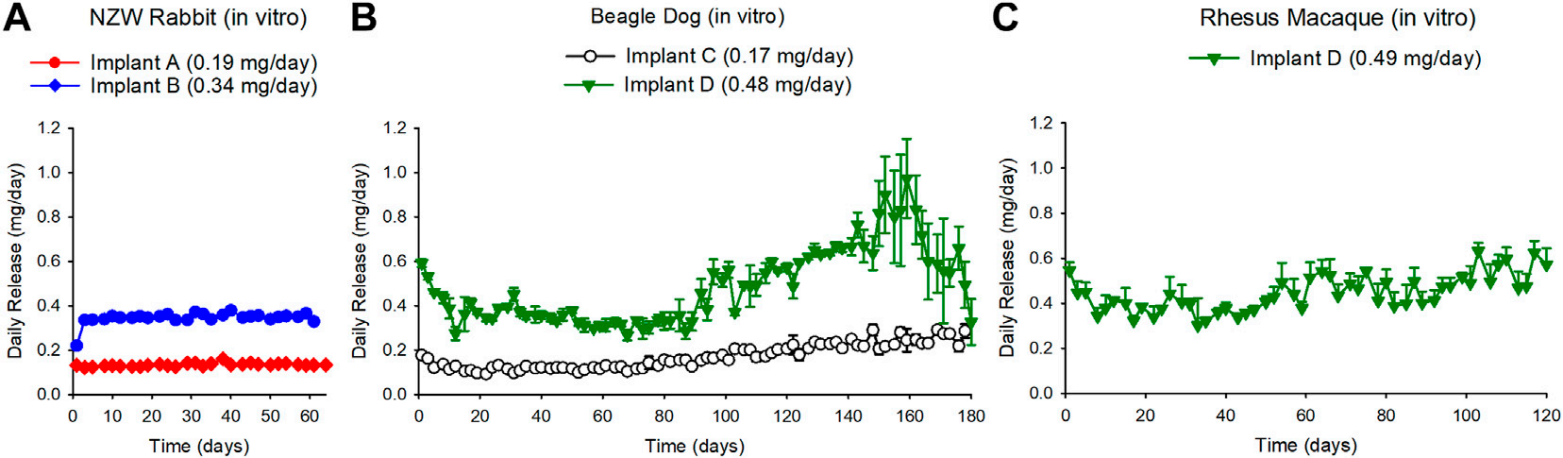
**(A)** Daily release rates of TAF from *in vitro* implants run in parallel with the *in vivo* study in NZW rabbits resulted in TAF release rate of 0.19 + 0.07 mg/day and 0.34 + 0.07 mg/day for the Implant A and the Implant B in the NZW rabbit corresponding groups, respectively. **(B)** Daily release rates of TAF from *in vitro* implants that were run in parallel with the *in vivo* study in beagle dogs resulted in a TAF release rate of 0.17 + 0.06 mg/day and 0.48 + 0.17 mg/day for the Implant C and the Implant D beagle dog corresponding groups, respectively. **(C)** Daily release rates of TAF from *in vitro* implants that were run in parallel with the *in vivo* study in rhesus macaque resulted in a TAF release rate of 0.49 + 0.16 mg/day for Implant D. Each data point represents the average + standard deviations.

*In vitro* studies performed in tandem with the *in vivo* study in NZW rabbits demonstrated sustained *in vitro* release of TAF salt from the implants with zero-order kinetics over the course of 63 days for Implant A and Implant B (Figure 2, Panel a). The overall release rates for Implant A and Implant B were 0.19 ± 0.07 and 0.34 ± 0.07 mg/day, respectively. The residual drug remaining within the used implants after the *in vitro* studies indicated that 18 and 25% of TAF_salt_ was released over the 63-days study for Implant A and Implant B, respectively (Supplementary Table S1). The average ±standard deviation (SD) chemical purity of TAF from the used implants was 91 ± 0.8% and 90.2 ± 0.8% for Implant A and Implant B, respectively (Supplementary Table S2).

#### 3.1.2 *In vitro* Studies Performed in Parallel to the Beagle Dog Study

The *in vitro* studies performed in tandem with the *in vivo* study in beagle dogs demonstrated release of TAF base from the implants for 180 days from Implant C and the Implant D (Figure 2, Panel b). The sustained release for Implant D lasted approximately to day 90 after which the release rate continued to increase up to day 160 reaching a release rate of 0.86 ± 0.07 mg/day between days 147–161 before decreasing (Figure 2, panel b). The overall release rate for Implant C and Implant D was 0.17 ± 0.06 and 0.48 ± 0.17 mg/day, respectively. The chemical purity of TAF within the implant at day 180 was 85.3% (85, 85.4 and 85.6%) for Implant C and 62 and 71% for Implant D in the dog study (Supplementary Table S2).

#### 3.1.3 *In vitro* Studies in Parallel to the Rhesus Macaque Study

The *in vitro* release of TAF base from the implants that were monitored in parallel to the rhesus macaque *in vivo* study demonstrated sustained release of TAF for Implant D over the course of 135 days (0.49 ± 0.16 mg/day) (Figure 2, Panel C). The chemical purity of TAF within the reservoir was 74.6 ± 9.5% at day 135 (Supplementary Table S2).

### 3.2 *In vivo* Studies: Pharmacokinetics

#### 3.2.1 Plasma TAF

Plasma TAF and non-compartmental analysis (NCA) are reported in Supplementary Tables S3, S5. In the NZW rabbit study, the median Cmax (±SD) concentration of TAF in plasma was maintained at 0.17 (±0.33), 0.24 (±0.05) and 0.64 (±0.4) ng/ml for Implant A, Implant B, and Implant B [2x] groups through Day 63, respectively (Supplementary Table S3). Within the dose range of 0.15–0.84 mg/day, plasma concentrations of TAF did not increase in proportion to the dose, either in terms of C_max_ or AUC_(0-t)._ In the dog studies, the median Cmax (±SD) levels of TAF in plasma were maintained at 0.3 (±0.06) and 1.0 (±2.8) ng/ml for Implant C and Implant D, respectively. Similar to the NZW rabbit studies, the plasma concentrations of TAF did not increase proportionally to the dose, within the range of 0.29–0.48 mg/day, either in terms of C_max_ or AUC_(0-t)_ (Supplementary Table S5). TAF was not quantifiable in plasma samples collected in the rhesus macaque study.

#### 3.2.2 Plasma TFV

Plasma TFV and NCA estimates are reported in Supplementary Tables S4, S6. As expected, TFV concentrations in plasma were stable throughout the studies. In the rabbit study, the median Cmax (±SD) concentrations of TFV in plasma were 3.6 (±2.9), 4.2 (±0.66), and 11.9 (±3.3) ng/ml for Implant A, Implant B, and Implant B [2X] groups through Day 63, respectively (Supplementary Table S4). Similar to the plasma exposure to TAF, exposure to plasma TFV was not dose-proportional both in terms of C_max_ and AUC_(0-t)_ (Supplementary Table S4). In the dog study, median (±SD) plasma TFV concentrations were maintained at 4.6 (±1.5) and 19.4 (±3.3) ng/ml for Implant C and Implant D, respectively. Exposure to TFV did not increase in proportion to the dose in the dose range 0.29–0.48 mg/day either in terms of C_max_ or AUC_(0-t)_ (Supplementary Table S6). TFV was not quantifiable in plasma samples collected in the rhesus macaque study.

#### 3.2.3 TFV-DP in PBMCs

**FIGURE 3:**
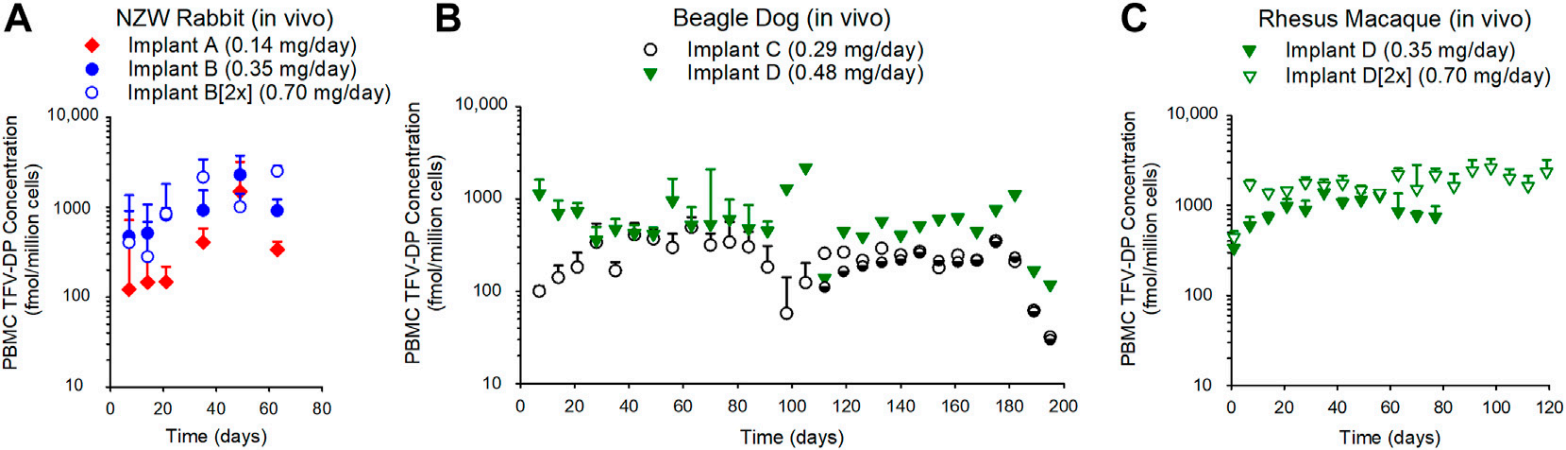
The subcutaneous implantation of TAF implants in NZW rabbit **(A)**, beagle dog **(B)**, and rhesus macaques **(C)** maintained TFV-DP in PBMCs over the duration of 63–182 days. **(A)** The median (IQR) of PBMC TFV-DP in the rabbit study for the Implant A (*n* = 5), Implant B (*n* = 5), and Implant B [2x] (*n* = 5) groups were 307 (218–353), 944 (852–1050), 1129 (804–1841) fmol/10^6^ cells, respectively. **(B)** The median (IQR) of PBMC TFV-DP concentrations in the dog study for Implant C (*n* = 6) and Implant D (*n* = 6) groups were 216 (152–303) and 502 (415–726) fmol/10^6^ cells, respectively. **(C)** The median (IQR) of PBMC TFV-DP in the rhesus macaque study for Implant D (*n* = 3), and Implant D [2x] (*n* = 3) groups were 927 (877–948) and 1698 (1561–1834) fmol/10^6^ cells, respectively. Each data point represents the median + standard deviations. In the dog study **(B)**, the remaining implants (Implant C [*n* = 2 of 3 implants] and Implant D [*n* = 1 of 3 implants]) were retrieved at day 182 and TFV-DP concentrations were determined during the 2-weeks washout.

The concentration of TFV-DP in PBMCs over time is plotted in Figure 3. TFV-DP concentrations in PBMCs were sustained above 100 fmol/10^6^ cells in all animals over 3–6 months. For all treatment groups, TFV-DP concentrations were quantifiable at 7 days post implantation. Median (IQR) TFV-DP concentrations were 307 (218–353), 944 (852–1050), and 1129 (804–1841) fmol/10^6^ cells for Implant A, Implant B, and Implant B [2x] groups through 63 days in the NZW rabbit study, respectively (Figure 3, Panel A). In the 6-month dog study, median (IQR) TFV-DP concentrations were 216 (152–303) and 502 (415–726) fmol/10^6^ cells for Implant C and Implant D, respectively (Figure 3, Panel B). Due to the premature failures of Implant C at day 115 and Implant D at day 90, a better reflection regarding the median (IQR) TFV-DP concentrations in the dog study are 289 (162–350) fmol/10^6^ cells for Implant C through day 115 and 505 (415–726) fmol/10^6^ cells for Implant D through day 90. Lastly, in rhesus macaques, median (IQR) TFV-DP concentrations were 927 (877–948), and 1698 (1561–1834) fmol/10^6^ cells for the Implant D, and Implant D [2x] groups, respectively (Figure 3, Panel C).

#### 3.2.4 TFV-DP Concentrations in the Mucosal Tissues of NZW Rabbits

**FIGURE 4:**
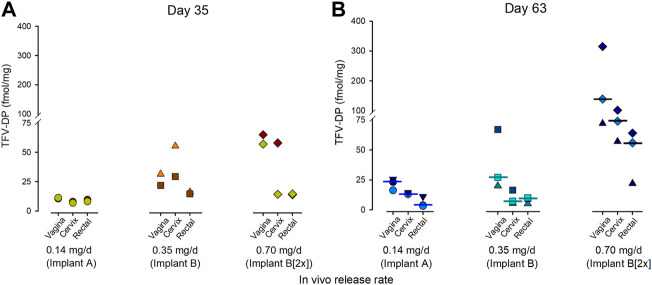
Concentrations of TFV-DP in the vaginal, cervical, and rectal tissues resulting from a TAF implant lasting 35 days **(A)** or 63 days **(B)** in the NZW rabbit. The data point corresponds to individual animals from each treatment group. The bar indicates the median TFV-DP concentrations.

The subcutaneous TAF implants loaded the rectal, cervical, and the vaginal tissues with TFV-DP. By day 35, TFV-DP concentrations were detected in all tissues with the highest concentrations in the vaginal and cervical tissues when compared to the concentrations in the rectal tissue (Figure 4A). The concentration of TFV-DP within tissues continued to increase over time resulting in median TFV-DP concentrations at day 63 of 110 fmol/mg, 75 fmol/mg and 55 fmol/mg in the vaginal, cervical, and rectal tissues, respectively (Figure 4B).

### 3.3 *In vivo* Performance and Histological Analysis of Tissue Surrounding the TAF Implants

In the NZW rabbit studies, all TAF implants (Implant A) were successfully retrieved at the predetermined study end date of 63 days. In the beagle dog studies, the premature retrieval of TAF implants occurred in 25% (3/12) of animals during the study, due to adverse clinical observations. For example, one dog with Implant C, and two dogs with Implant D underwent early removal of implants before the end of the 6-months study due to skin abscesses near the implantation site that failed to respond to topical ointments. Upon retrieval of the implants, it was noted that these implants were fractured and most likely were fractured prior to retrieval resulting in the clinical observations. In the macaque study, all of the TAF implants (Implant D) were retrieved at the predetermined study end date of 84 or 140 days.

We observed potential adverse effects in both the TAF base and TAF salt implants underneath the skin and surrounding tissues and became more conspicuous the longer the implants remained in the animals. In the rabbit studies, skin biopsies near the implantation site were submitted for histological analysis and to determine the concentrations of TFV-DP. Overall, inflammation and thickening of the epidermis, dermis, and subcutis were noted in 33% (3/9) of animals. The subcutaneous reaction was characterized by granulomatous inflammation, neovascularization, and proliferation of the subcutaneous connective tissue surrounding the implant. The subcutaneous granulomatous tissue in most of these animals had formed a fibrous capsule surrounding the implant. Even in the presence of fibrous capsule formation, the observed plasma concentrations indicated that the release of TAF from the implants was not adversely affected (Figure 3, Panel A). It was also noted tissues near the implant had a central area of necrotic debris. Interestingly in the rabbit study, the skin tissues with localized reactions contained higher concentrations of TFV-DP when compared to concentrations of TFV-DP in uncompromised skin tissue. (Supplementary Table S7).

In the dog studies, microscopic changes were present in 17/32 of skin sections near the placebo implant and in 26/28 skin sections near the TAF implants. The majority of microscopic changes at sites near the placebo implant were minimal to mild and were considered within the range of expected changes at a healing/healed subcutaneous implant site (data not shown). In the sections taken at day 91 and day 182, the sites near the TAF implant had minimal to marked lymphocytes, plasma cells, polymorphonuclear cells, and/or histiocytes/macrophages, surrounded by bands of fibrosis (ranging up to severe) (Supplementary Table S8). In nearly all of the sections harvested (26/28), a central core of eosinophilic material was present comprising lymphocytes, plasma cells, polymorphonuclear cells, and/or histiocytes/macrophages surrounded by bands of fibrosis (Supplementary Table S8). Where inflammation was present, the severity scores were similar to those from the Day 91 time-point, thus indicating minimal to no resolution of inflammation at the site. It was noted that necrosis was present in all animals. The surgical removal of the implant prior to histopathology prevented definitive determination of whether tissue reactions were due primarily to the implant itself or the TAF within the implant. However, the reduced inflammation and fibrosis in placebo implant sites strongly suggest that the inflammation in active implant sites was primarily due to the TAF component of the test article.

**FIGURE 5:**
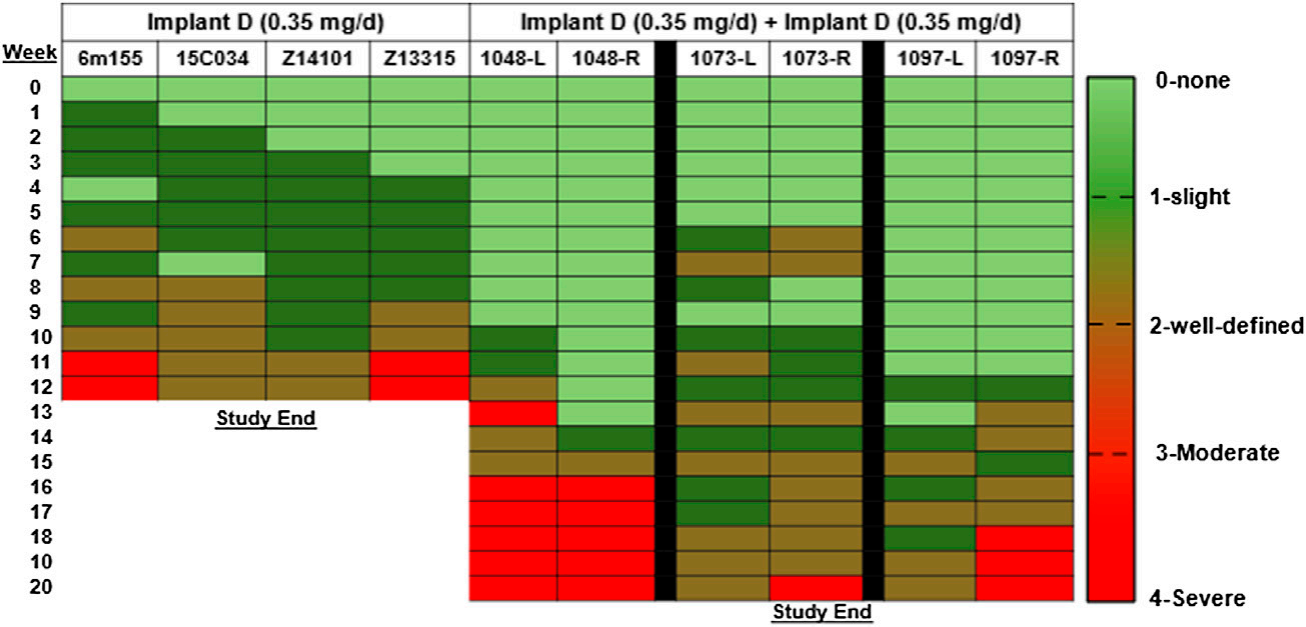
Visual assessment of local skin reactions in the rhesus macaques using the Draize assessment.

In the rhesus macaques, a modified Draize scale was used to assess erythema and swelling visually around the implant. Overall, the local skin reactions to the TAF implants were assessed to be slight to well-defined outcomes (Figure 5). Local skin reactions were absent (grade 0) in 23% in the Implant D group and 50% in the Implant D [2x] group. Mild reactions (grade 1–2) were observed in 63% of the Implant D group and in 40% of the Implant D [2x] group of observations. Moderate to severe reactions (grade 3–4) occurred in 14 and 10% of observations in the Implant D and Implant D [2x] groups, respectively. Skin biopsies taken at day 77 and 118 near the implant from animals in Implant D and Implant D [2x] (respectively) (Table 2) exhibited similar inflammatory markers (lymphocytes, plasma cells, polymorphonuclear cells, and/or histiocytes/macrophages, surrounded by bands of fibrosis) as those reported in the dog (Supplementary Table S8).

**TABLE 2:** Local reactivity in rhesus macaques.

Rhesus macaques	Total Possible	Implant D (0.35 mg/day)	Implant D [2x] (0.7 mg/day)
Observations^a^	Score	84 days	140 days
Local reactivity Score			
Grade 0		23% (14/62)	50% (57/114)
Grade 1-2		63% (39/62)	40% (46/114)
Grade 3-4		14% (9/62)	10% (11/114)
Fibrosis^b^	5	1.3 ± 0.6	1.7 ± 1.2
Inflammatory cells			
Polymorphonuclear cells	5	3.3 ± 1.2	1.3 ± 0.6
Lymphocyte cells	5	2.7 + 0.6	2.3 ± 0.6
Macrophages	5	2.7 ± 1.2	2 + 1
Plasma Cells	5	2.3 ± 0.6	2 ± 1

a Observations were graded as the following: 0 = None; 1 = Slight; 2 = Well-defined; 3 = Moderate; 4 = Severe.

b Scores recorded for lymphocytes, plasma cells, histiocytes/macrophages, polymorphonuclear cells, fibrosis, proliferating fibroblasts, and acute hemorrhage in the subcutaneous tissue (0–5 scale). Mean (+SD) score from the four dermal sections (cranial, medial, caudal, lateral) submitted for histopathology and rated as the following: 0 = None; 1 = Rare; 2 = Moderate/scattered; 3 = Moderate; 4 = Moderate to marked; 5 = Abundant/extensive with 5 being the highest maximum score.

Overall, the severity of local skin reactions and inflammation was greatest between months 2–3 post implantation and then plateaued for the remainder of the study (Table 2 and Supplementary Table S8). The TAF implant sites revealed moderate to marked deep dermal inflammation and subcutaneous necrosis in all species, whereas the placebo implant sites exhibited minimal to mild inflammation which was considered unremarkable and within range of expected changes at the healing/healed site.

## 4 Discussion

The primary endpoints of these preclinical studies were to evaluate the subcutaneous delivery of TAF from biodegradable PCL implants and to assess the local histological response in three different animal species. The concentration of TFV-DP in PBMCs is recognized as a clinical target for HIV PrEP with FTC/TDF, (Anderson et al., 2012; Gunawardana et al., 2015; Hendrix et al., 2016), and was therefore used as a benchmark in evaluating the performance of the TAF implant. The concentration of TFV-DP in PBMCs associated with effective HIV PrEP is estimated at 24–48 fmol/10^6^ cells in humans, (Gunawardana et al., 2015), and these studies aimed to exceed these levels using our subcutaneous implant. Incremental changes to the specifications of the PCL implants (form of TAF, excipients, wall thicknesses, and polymer formulation), resulted in sustained concentrations of TFV-DP in PBMCs in NZW rabbits, beagle dogs, and rhesus macaques indicating the PCL implants effectively delivered TAF over the course of 63 days (in rabbits) to 180 days (in dogs). By adjusting the parameters of the implant, the release rate was modified to deliver TAF at different dose levels which were demonstrated by the dose-related PK across all species (Figure 3). Upon removal of the PCL TAF implants from the beagle dogs at day 180, TFV-DP concentrations in PBMCs decreased over 2 weeks (Figure 3B; Supplementary Figures S4, S5B). It should be noted the plasma concentrations in the dog study were only monitored for 14 days after PCL implant removal, which may be an insufficient duration for TAF washout from the animal. As an exception, Implant D was removed on day 105 from an animal due to localized skin reactions, but blood sampling continued (Supplementary Figure S5A). Weekly monitoring revealed a steady decline in TFV-DP concentrations and required 5 weeks to reach unquantifiable concentrations, indicative of TAF washout.

In all species, median TFV-DP concentrations exceeded the concentration of the predicted target in humans (24–48 fmol/10^6^ cells (Gunawardana et al., 2015)) and were on the order of 6 (Implant A in the rabbit) to 35 times (Implant D [2x] in the rhesus macaque) higher. While preventative benchmarks for regimens of TAF alone have not been defined, the implant is readily adjustable to increase or reduce the daily release rates of TAF to align with desirable preventative targets in humans. Implants releasing TAF at 0.7 mg/day in the rhesus macaques resulted in a TFV-DP concentration in PBMCs of 1674 fmol/10^6^ cells, which is a concentration associated with nearly 93% protection against vaginal SHIV infection in pigtail macaques receiving an clinical equivalent oral dose of TAF (Massud, 2021). The TFV-DP concentrations in rabbit vaginal tissues were approximately 3-fold higher when compared to TFV-DP concentrations in the rectum of female NZW rabbits (Figure 4B).

While assessing the PBMC TFV-DP concentrations in our animal studies, we also noticed differences between the performance of our PCL TAF implants and other subcutaneous TAF implants (Gunawardana et al., 2015; Chua et al., 2018; Su et al., 2020). In the present study, the TFV-DP concentrations in the PBMCs exceed concentrations previously reported with TAF implants with similar release rates (Gunawardana et al., 2015; Chua et al., 2018; Su et al., 2020). In NZW rabbits, for example, the median TFV-DP concentrations in PBMCs were 68 fmol/10^6^ cells and 218 fmol/10^6^ cells for subcutaneous implants with TAF release rates of 0.13 mg/day and 0.78 mg/day, respectively. (Su et al., 2020) With our release rates in NZW rabbits of 0.15 mg/day (Implant A) and 0.84 mg/day (Implant B [2x]), the median TFV-DP concentrations were 307 fmol/10^6^ cells and 1129 fmol/10^6^ cells, respectively (Figure 3A). In a previously reported beagle dog study, a release rate of approximately 1.08 mg/day yielded a median TFV-DP concentration in PBMCs of 511.8 fmol/10^6^ cells over 35 days (Gunawardana et al., 2015) whereas in our dog study at 35 days, the median TFV-DP concentration of 470 fmol/10^6^ cells was achieved with Implant D releasing TAF at a rate approximately 50% lower (i.e., 0.48 mg/day) (Figure 3B). The *in vitro* release rates of Implant D increase at approximately 80 days (Figure 2B), which was likely caused by the hydrolytic instability of TAF. An increase in the production of TAF degradants can result in a higher causes an increase of water ingress into the implant and subsequently increases the release rates of and ultimately higher TAF release rates (Li et al., 2020). Finally, in rhesus macaques, the median concentrations of TFV-DP of 394 and 643 fmol/10^6^ cells were reported from implants with TAF release rates of 0.39 and 0.78 mg/day, respectively (Su et al., 2020) whereas in our rhesus macaque studies, release rates of 0.35 mg/day (Implant D) and 0.70 mg/day (Implant D [2x]) resulted in median PBMC TFV-DP concentrations of 869 and 1674 fmol/10^6^ cells, respectively.

Unlike the other investigations that used TAF provided by Gilead Sciences, TAF hemifumarate and TAF free base used in the present preclinical studies were sourced from a commercial chemical supplier, BOC Sciences. The certificate of analysis provided by the company demonstrated that these drug substances were fully characterized and were of comparable purity. Under identical experimental procedures, *in vitro* studies comparing TAF hemifumarate and TAF free base provided by Gilead Sciences to those sourced from BOC Sciences indicated nearly identical release rate characteristics from our PCL implants (data not shown). It is unlikely that differently sourced TAF could result in differences in TFV-DP concentrations.

The potential for drug dumping due to premature failure of the PCL implants might cause the higher concentrations of TFV-DP in PBMCs and skin tissues reported here. It is evident that Implant C and Implant D in the beagle dog suffered from integrity issues, where the implants were bent or compromised likely due to the thinner wall configurations (i.e., 100 µm). Only 50% (3/6) of the implants completed the 6-months dog study. However, the TFV-DP concentration in the animal with the known failure of Implant D at day 105 in the dog study was 363 fmol/10^6^ cells, which was markedly lower than the concentrations in rabbits implanted with Implant B (944 fmol/10^6^ cells) and in rhesus macaques with Implant D (869 fmol/10^6^ cells). Unlike the implants used in the 6-months dog studies, majority of the implants in the rabbit and macaque studies remained largely intact for the entire study period suggesting the failure of the implants in the dog studies did not contribute to the comparatively high concentrations of TFV-DP observed in these present studies when compared to other TAF implants (Gunawardana et al., 2015; Chua et al., 2018; Su et al., 2020).

One difference that exists with our implant is the drug formulation, wherein TAF is combined with an oil excipient (e.g., castor oil or sesame oil) that can facilitate the solubilization of the TAF, enhance the drug dissolution rate, (Li et al., 2021a), and optimize the stability of TAF *in vivo* (Supplementary Table S2). Additional studies are required to further understand how potential effects of these excipient-based formulations affect the performance *in vivo*, such as an increase in the concentrations of TFV-DP within PBMCs.

While we are encouraged with the potential of this biodegradable PCL implant to sustainably release TAF and produce high TFV-DP concentrations over 3–6 months, challenges exist. The local skin reactions reported near the implantation site in NZW rabbit, beagle dog, and rhesus macaques align with other investigators who report similar histopathological findings with subcutaneously administered TAF via an in-dwelling catheter (Romano et al., 2021) or a non-degradable implant (Su et al., 2020). Unlike the TAF implants, the placebo implants did not elicit a subcutaneous reaction indicating implants containing castor oil or sesame oil were well tolerated. At TFV-DP concentrations in PBMCs much lower than those reported here, inflammation and localized skin reactions were observed near the implantation site of NZW rabbits and rhesus macaques (Su et al., 2020). Additionally, dog studies using TAF implants have alluded to possible adverse outcomes such as edema and erythema near the implantation site (Gengiah et al., 2019). In our rabbit study, local reactivity outcomes were primarily infiltration of lymphocytes, macrophages, and granulocytes and, interestingly, were associated with high TFV-DP concentrations in tissues near the TAF implants (Supplementary Table S7). A more comprehensive histopathology assessment was performed in the beagle dogs, and the predominant immune cells present in tissues sections taken near the implantation site were lymphocytes, plasma cells, polymorphonuclear cells, histiocytes/macrophages. The histopathological scores between tissues taken at mid-study and the time of implant removal indicated little to no resolution in the inflammatory response over time (Supplementary Table S8). Unlike the tissues near the placebo implant, necrosis was present in tissues of all animals implanted with the TAF implant. Additionally, 50% (3/6) of the TAF implants were removed earlier than planned due to localized skin reactions. The Draize assessment used in the rhesus macaque study demonstrated that the severity of clinically visible skin reactions near the TAF implant sites increased with time (Figure 5). Upon retrieval of the implants, the underlying tissues near the TAF implant were noted as necrotic in some of the macaques. The reason for the subcutaneous reactions that occurred in nearly all types of TAF implants under development is largely unknown. Subcutaneous reactions may be due to local tissue exposure to TAF, TAF-related metabolites such as [(R)-9-(2-phosphonome-thoxypropyl)adenine (PMPA) monoamidite and TFV), and/or TFV-DP. Importantly, all animals were returned to stock colony and for the dogs and macaques, the localized skin reactions started to resolve within 2 weeks following the removal of the TAF implant.

In summary, we have demonstrated that a biodegradable PCL implant releasing TAF produces sustained TFV-DP concentrations in PBMCs in NZW rabbits, beagle dogs, and rhesus macaques over a duration of 3–6 months. By delivering high TFV-DP concentrations in PBMCs, these data show the potential of a TAF implant as an effective device for HIV prevention in humans. The high concentrations of TFV-DP in tissues near the TAF implant might suggest optimizing the location of a TAF implant to be closer to the target tissues to deliver an efficacious TAF dose for HIV PrEP in humans, formulating TAF with other API to reduce dosage needed, or exploring other potent APIs for HIV PrEP in our platform. Evidence has indicated that penetration of NNRT inhibitors like TAF in adipose tissue are limited when compared to integrase inhibitors suggesting the ability of TAF to completely suppress viral replication might be impaired (Couturier et al., 2018). While we presented TFV-DP concentrations in tissue matrices relevant to HIV (genital tissues, PBMCs and tissues near the implantation site), we didn’t collect tissue reservoirs pertinent to the continued maintenance of viral suppression. Future preclinical implant studies that include adipose tissue and lymph nodes are required to elucidate the penetration and effectiveness of antiretroviral drugs in cellular and anatomic viral reservoir sites.

This manuscript details the preclinical studies of a biodegradable reservoir-style implant for delivery of TAF, which informed key attributes of the implant and supported progressive development efforts. Additional work is still required to align the biodegradation timeframe of the PCL with drug delivery profile to minimize the persistence of the polymer in the subcutaneous space after the drug depletes. Modifications in wall thickness, drug formulations (i.e., form of TAF and excipient choice), and Mw of PCL ultimately produced an implant with targeted dosing profiles. The preclinical evaluation of safety and PK showed sustained concentrations of TFV-DP in PBMCs over 100 fmol/10^6^ cells in all animal species (NZW rabbit, beagle dog, rhesus macaque) and effectively delivered TAF for 3–6 months. For all the iterations of the implant, the delivery of TAF within the subcutaneous space resulted in adverse effects proximal to the implant, including dermal inflammation and necrosis across all species. Despite these adverse events, the implant as a platform technology performed as designed and achieved a constant drug release profile, supporting the continued development of this drug delivery approach.

## Data Availability

The raw data supporting the conclusions of this article will be made available by the authors, without undue reservation.
