# Supplementary material for: Comparative Pharmacokinetics and Local Tolerance of Tenofovir Alafenamide (TAF) From Subcutaneous Implant in Rabbits, Dogs, and Macaques
Source: Front Pharmacol. 2022 Jul 19;13:923954. doi: 10.3389/fphar.2022.923954 (PMC9343794; doi:10.3389/fphar.2022.923954)
Supplement: Supplementary file 1 [file DataSheet1.docx]

Supplementary Material


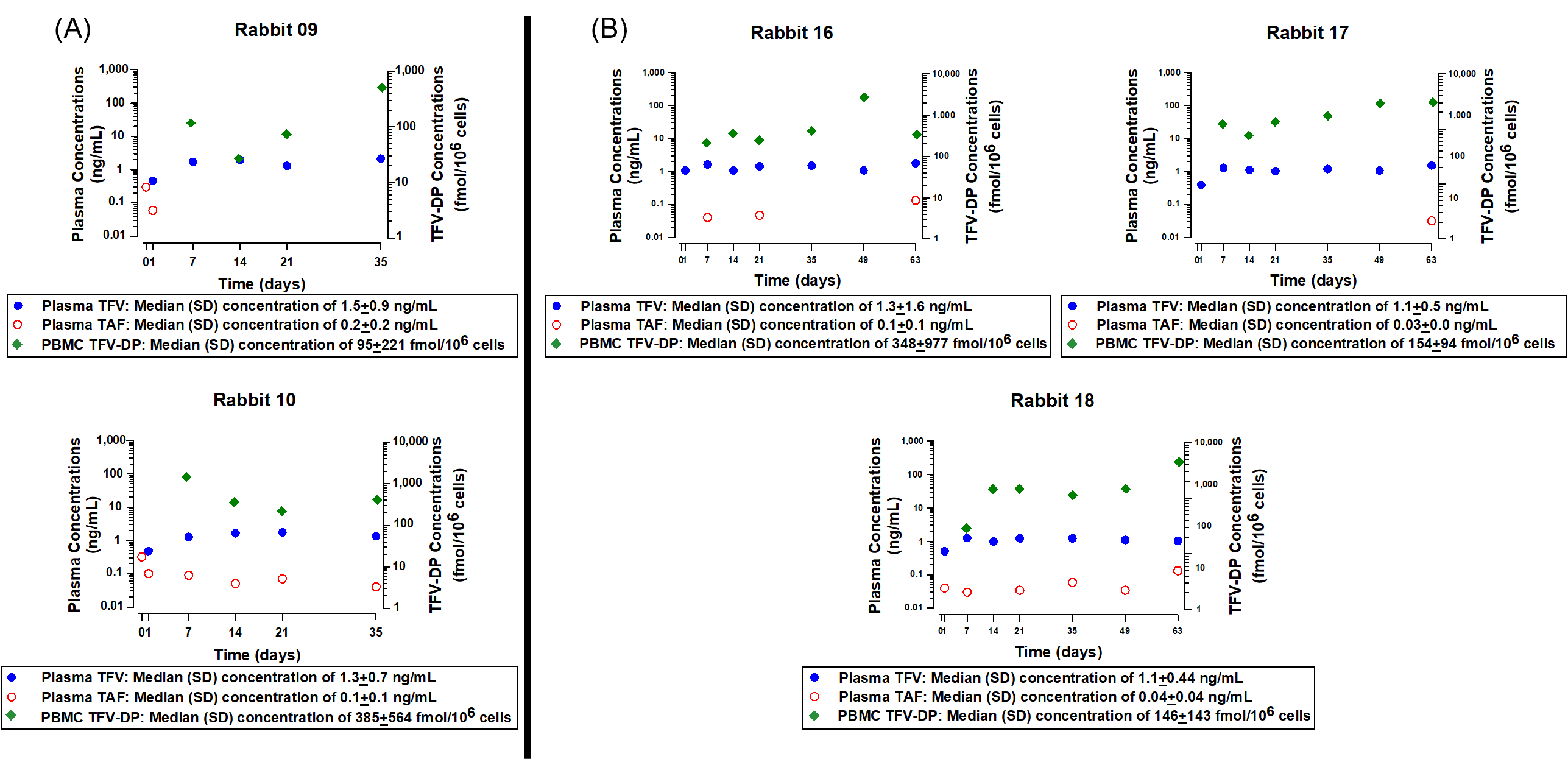


**Figure S1.** Individual pharmacokinetic profiles of plasma TAF, plasma TFV and PBMC TFV-DP in rabbits inserted with Implant A over a study duration of 35 days **(A)** or 63 days **(B)**. TAF (red), TFV (blue) and TFV-DP (green) were analysed at the indicated time points. Median (+SD) TAF, TFV, and TFV-DP concentrations are provided in summary box for each animal.

**
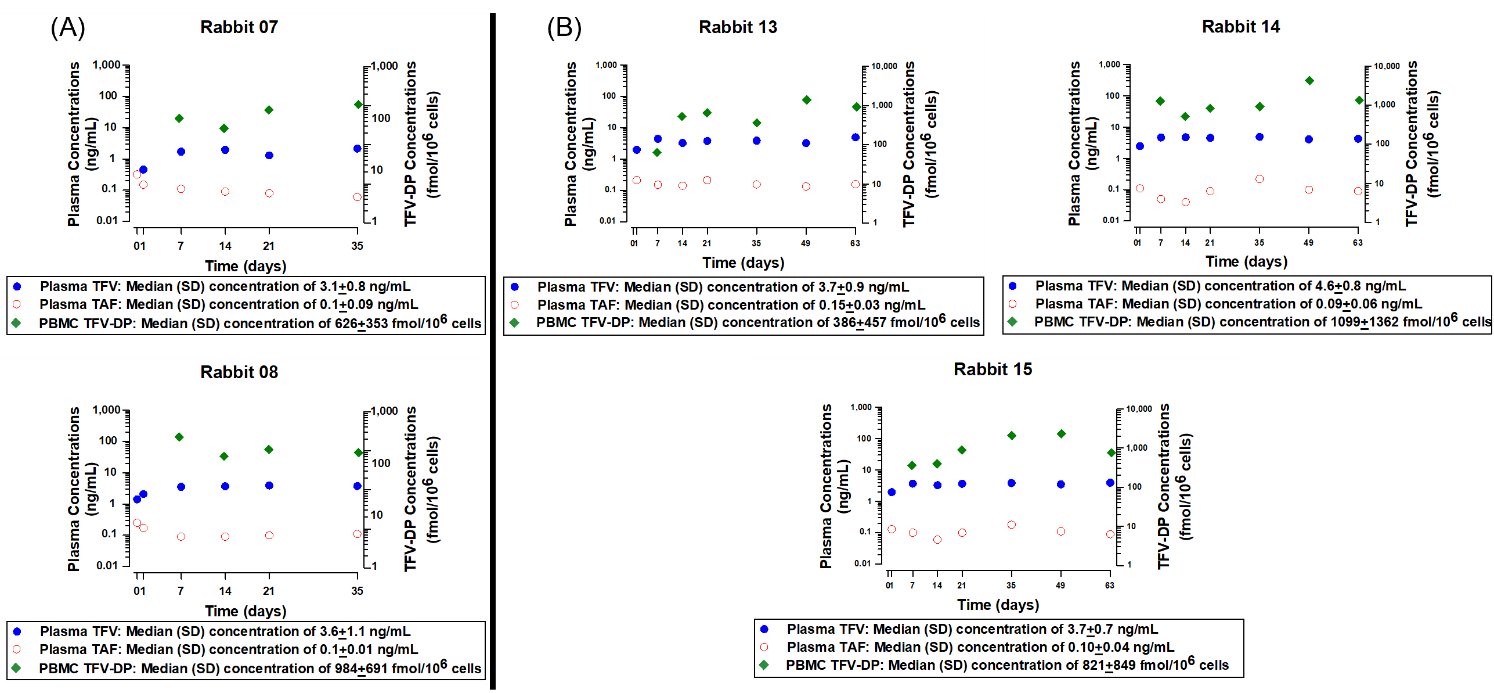
**

**Figure S2.** Individual pharmacokinetic profiles of plasma TAF, plasma TFV and PBMC TFV-DP in rabbits inserted with Implant B over a study duration of 35 days **(A)** or 63 days **(B)**. TAF (red), TFV (blue) and TFV-DP (green) were analysed at the indicated time points. Median (+SD) TAF, TFV, and TFV-DP concentrations are provided in summary box for each animal.

**
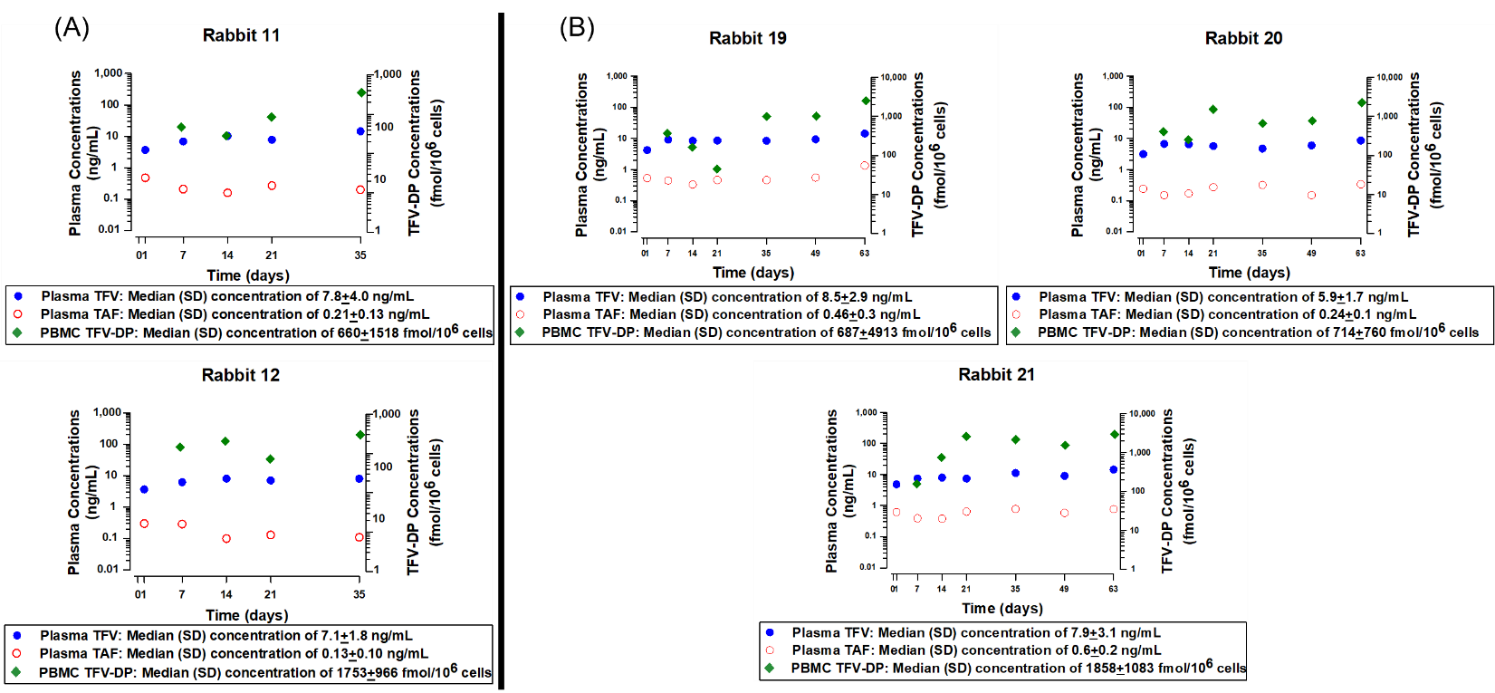
**

**Figure S3.** Individual pharmacokinetic profiles of plasma TAF, plasma TFV and PBMC TFV-DP in rabbits inserted with Implant B[2x] over a study duration of 35 days **(A)** or 63 days **(B)**. TAF (red), TFV (blue) and TFV-DP (green) were analysed at the indicated time points. Median (+SD) TAF, TFV, and TFV-DP concentrations are provided in summary box for each animal.

**
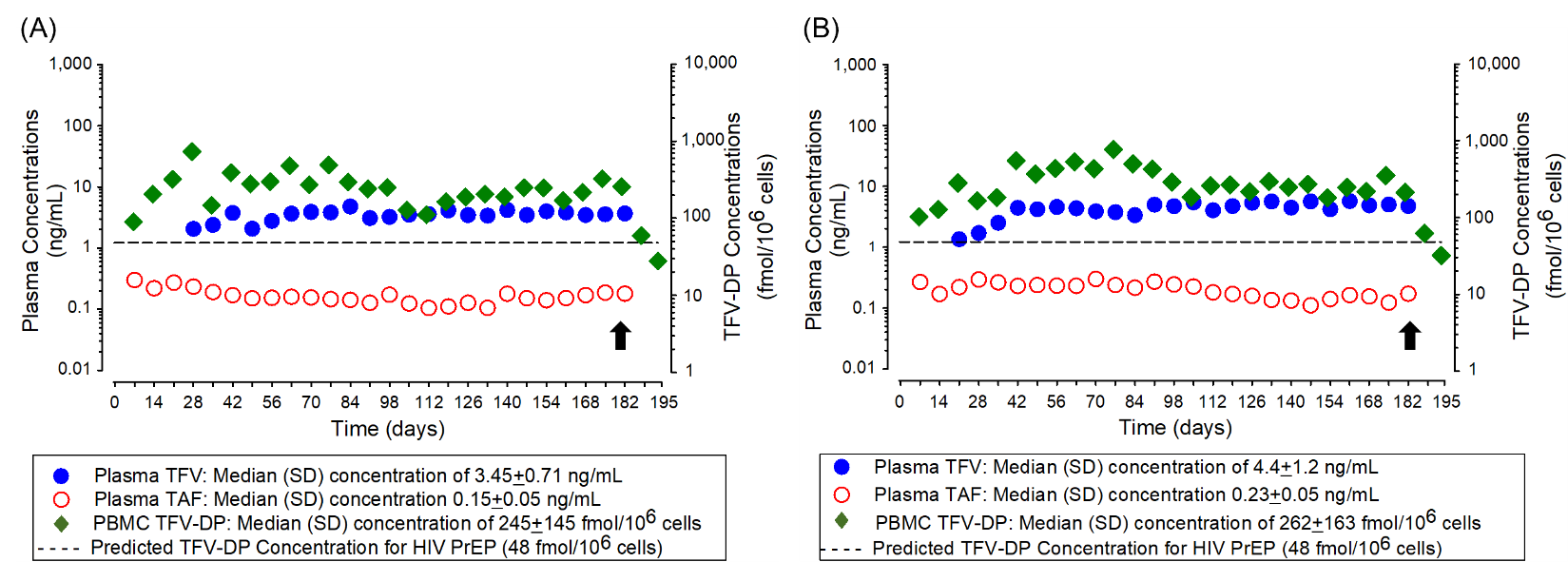
**

**Figure S4.** Individual pharmacokinetic profiles of plasma TAF, plasma TFV and PBMC TFV-DP in dogs implanted with Implant C over a study duration of 182 days in animal #1004 **(A)** and animal # 1006 (**B)**. TAF (red), TFV (blue) and TFV-DP (green) were analysed at the indicated time points. On day 182, implants were retrieved **(denoted by the black arrows)** and plasma and PBMCs were collected during the 2-week washout period. Median (+SD) TAF, TFV, and TFV-DP concentrations are provided in summary box for each animal. Horizontal dotted line represents preventative target concentrations for HIV PrEP.

**
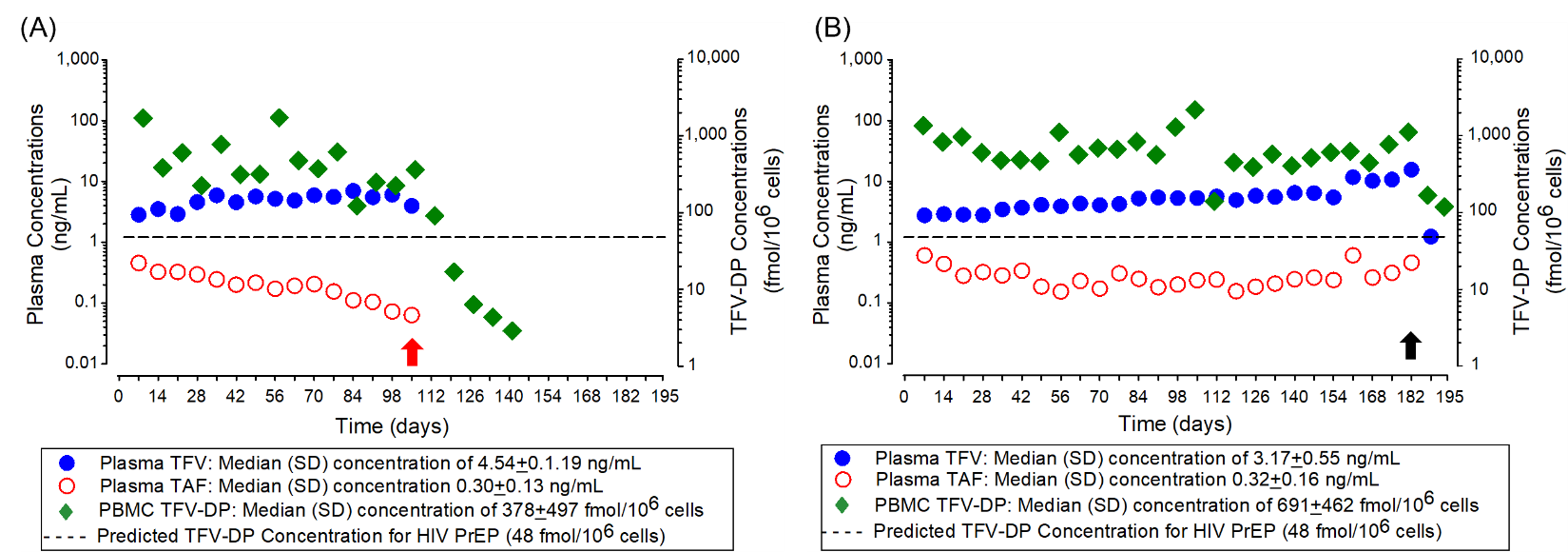
**

**Figure S5.** Individual pharmacokinetic profiles of plasma TAF, plasma TFV and PBMC TFV-DP in dogs inserted with Implant D over a study duration of 105 days in animal #1010 **(A)** and 182 days in animal #1011 **(B)**. TAF (red), TFV (blue) and TFV-DP (green) were analysed at the indicated time points. On day 105, the implant in animal #1010 was removed due to local skin reactions **(denoted by the red arrow)** but plasma and PBMCs were collected for an additional 5 weeks to examine a longer washout than the typical 2-week washout period. On day 182, the Implant in animal ID#1011 was retrieved **(denoted by the black arrow)** and plasma and PBMCs were collected during the 2-week washout period. Median (+SD) TAF, TFV, and TFV-DP concentrations are provided in summary box for each animal. Horizontal dotted line represents preventative target concentrations for HIV PrEP.

| **Implant** | **Study Arm** | **TAF Loaded (mg)** | **Residual TAF**  **(mg)** | **Estimated Release Rate**  **(mg/day)** |
| --- | --- | --- | --- | --- |
| Implant A | 63d In vitro | 116.1 | 96.2 | 0.15 |
|  | 63d In vitro | 114.5 | 87.63 | 0.29 |
|  | 63d In vitro | 116.9 | 100.3 | 0.12 |
| Implant B | 63d In vitro | 130.2 | 98.0 | 0.34 |
|  | 63d In vitro | 135.2 | 105.2 | 0.27 |
|  | 63d In vitro | 139.9 | 99.2 | 0.43 |

Table S1**.** UPLC analysis of residual TAF extracted from the reservoir of the implant after the in vitro release experiments in parallel to the NZW rabbit study.

| **Implant** | **Chemical Purity *In Vitro*** | **Chemical Purity *In Vivo*** |
| --- | --- | --- |
| **Implant A** | **(0.19 mg/day)** | **(0.14 mg/day)** |
| 63d | 91.0 | NA |
| 63d | 91.0 | NA |
| 63d | 92.4 | NA |
| **Implant B** | **(0.34 mg/day)** | **(0.35 mg/day)** |
| 63d | 91 | NA |
| 63d | 90.2 | NA |
| 63d | 89.4 | NA |
| **Implant C** | **(0.17 mg/day)** | **(0.29 mg/day)** |
| 90d | 95.3 | 96.9 |
| 90d | 95.4 | 97.6 |
| 90d | NA | 97.2 |
| 180d | 85.6 | 88.0 |
| 180d | 85.4 | 92.6 |
| 180d | 85.0 | 86.1 |
| **Implant D** | **(0.48 mg/day)** | **(0.48 mg/day)** |
| 90d | 93.8 | 96.1 |
| 90d | 94.0 | 96.4 |
| 90d | NA | 95.7 |
| 180d | 62.0 | 72.1 |
| 180d | 71.0 | NA |
| **Implant D** | **(0.49 mg/day)** | **(0.35 mg/day)** |
| 135d* | 80.4 | NA |
| 135d* | 82.2 | NA |
| 135d* | 61.2 | NA |
| 149d* | NA | 91.9 |
| 149d* | NA | 91.1 |

Table S2**.** TAF stability as expressed a % purity of the *in vitro* and *in vivo* excised implant devices. Purity as determined by UV-UPLC. In vivo release rates calculated by measuring the content of API in the implant after removal from the animal. *Data from rhesus macaques.

| **Dose** | **Estimated**  **Release Rate** | **Estimated**  **Dose Level** | **t_max_** | **C_max_** | **AUC_(0-t)_** |
| --- | --- | --- | --- | --- | --- |
| **Group** | **(mg/day)** | **(mg/kg)** | **(d)** | **(ng/mL)** | **(ng∙d/mL)** |
| Implant A | 0.14 | 0.06 | 38 | 0.17 (0.33) | 0.4 (0.2) |
| Implant B | 0.35 | 0.14 | 18 | 0.24 (0.05) | 6.3 2.8) |
| Implant B[2x] | 0.70 | 0.28 | 32 | 0.64 (0.4) | 20.4 (15.1) |
|  |  |  |  |  |  |

Table S3**.** Pharmacokinetics parameters (Median + SD) for TAF calculated from mean plasma concentrations-time of subcutaneously administration of TAF from the implants over the course of the 63-day rabbit study.

| Dose | **Estimated Release Rate** | **Estimated**  **Dose Level** | **t_max_** | **C_max_** | **AUC_(0-t)_** |
| --- | --- | --- | --- | --- | --- |
| **Group** | **mg/day** | **mg/kg** | **d** | **ng/mL** | **ng∙d/mL** |
| Implant A | 0.14 | 0.06 | 38 | 3.6 (2.9) | 117 (59.3) |
| Implant B | 0.35 | 0.14 | 25 | 4.2 (0.66) | 190 (72) |
| Implant B[2x] | 0.70 | 0.28 | 48 | 11.9 (3.3) | 413 (154) |

Table S4**.** Pharmacokinetic parameters (Median + SD) for TFV calculated from mean plasma-time of subcutaneously administration of TAF from the implants over the course of the 63-day rabbit study.

| **Dose** | **Estimated Release Rate** | **Estimated**  **Dose Level** | **t_max_** | **C_max_** | **AUC_(0-t)_** |
| --- | --- | --- | --- | --- | --- |
| **Group** | **(mg/day)** | **(mg/kg)** | **(d)** | **(ng/mL)** | **(ng∙d/mL)** |
| Implant C | 0.29 | 0.03 | 77 | 0.3 (0.06) | 17.9 (12.8) |
|  |  |  |  |  |  |
| Implant D | 0.48 | 0.04 | 74 | 1.0 (2.8) | 46.6 (190) |
|  |  |  |  |  |  |

Table S5**.** Pharmacokinetics parameters (Median + SD) for TAF calculated from mean plasma concentrations-time of subcutaneously administration of TAF from the implants in the dog study.

| Dose | **Estimated Release Rate** | **Estimated**  **Dose Level** | **t_max_** | **C_max_** | **AUC_(0-t)_** |
| --- | --- | --- | --- | --- | --- |
| **Group** | **mg/day** | **mg/kg** | **d** | **ng/mL** | **ng∙d/mL** |
| Implant C | 0.29 | 0.03 | 88 | 4.6 (1.5) | 283 (241) |
|  |  |  |  |  |  |
| Implant D | 0.48 | 0.04 | 43 | 19.4 (3.3) | 660 (395) |

Table S6**.** Pharmacokinetic parameters (Median + SD) for TFV calculated from mean plasma-time of subcutaneously administration of TAF from the implants in dog study.

| ~~Device ID~~ | **~~Original TAF~~**  **~~loading~~** | **~~Experimental~~**  **~~TAF recovered (mg)~~** | **~~TAF~~**  **~~released~~**  **~~(mg)~~** | **~~Estimated release rate in vivo~~**  **~~(mg/day)~~** | **~~Right/Left~~**  **~~Ratio~~** |
| --- | --- | --- | --- | --- | --- |
| ~~1097R~~ | ~~120.6~~ | ~~24~~ | ~~96.6~~ | ~~0.65~~ | ~~2.7~~ |
| ~~1097L~~ | ~~121.3~~ | ~~85~~ | ~~36.3~~ | ~~0.24~~ |  |
| ~~1048R~~ | ~~120.3~~ | ~~6~~ | ~~114.3~~ | ~~0.77~~ | ~~1.6~~ |
| ~~1048L~~ | ~~120.9~~ | ~~51.3~~ | ~~69.6~~ | ~~0.47~~ |  |
| ~~1073R~~ | ~~120.5~~ | ~~22~~ | ~~98.5~~ | ~~0.66~~ | ~~1.8~~ |
| ~~1073L~~ | ~~122.2~~ | ~~66.9~~ | ~~55.3~~ | ~~0.37~~ |  |

Table S7**.** Estimated release rates of TAF calculated from explanted implants from the rhesus macaques at day 149.

| NZW Rabbit | **TFV (ng/mg)** | | **TFV-DP (fmol/mg)** | | | **Histology** | | | |  |
| --- | --- | --- | --- | --- | --- | --- | --- | --- | --- | --- |
| **Implant**  **Animal ID** | **Active Implant** | **Placebo**  **Implant** | **Active Implant** | **Placebo**  **Implant** | | **Active**  **Implant** | | **Placebo Implant** | |  |
| **Implant A (0.14 mg/day)** | | |  | |  | |  | |  | |
| ID No.16 | 0.2 | BLQ | 6.2 | | BLQ | | Normal | | Normal | |
| ID No.17 | 0.1 | BLQ | 3.7 | | BLQ | | Normal | | Normal | |
| ID No. 18 | 0.1 | BLQ | **799** | | 4.2 | | **Infiltration of lymphocytes, macrophages and granulocytes** | | Normal | |
| **Implant B (0.35 mg/day)** | | |  | |  | |  | |  | |
| ID No. 13 | 1 | 0.3 | 964 | | 2 | | Normal | | Normal | |
| ID No. 14 | 0.5 | 0.3 | 2 | | BLQ | | Normal | | Normal | |
| ID No. 15 | 0.1 | 0.1 | **5556** | | 4.3 | | **Infiltration of lymphocytes, macrophages and granulocytes** | | Normal | |
| **Implant B[2x] (0.70 mg/day)** | | | | | | |  | |  | |
|  | **Active Implant** | **Active**  **Implant** | **Active Implant** | | **Active**  **Implant** | | **Active**  **Implant** | | **Active Implant** | |
| ID No. 19 | 0.5/1.3 | NA | **6623 & 6297** | | NA | | Normal | | **Infiltration of lymphocytes, macrophages and granulocytes** | |
| ID No. 20 | 0.1/0.5 | NA | 119 & 4652 | | NA | | **Infiltration of lymphocytes, macrophages and granulocytes** | | **Infiltration of lymphocytes, macrophages and granulocytes** | |
| ID No. 21 | 0.1/0.1 | NA | 394 & 5.4 | | NA | | Normal | | Normal | |

Table S7**.** TFV and TFV-DP concentrations (fmol/mg) in tissues near the implants and local reactivity outcomes in the NZW rabbits at Day 63.

| Beagle dog | **Total** | **Implant C**  **(0.29 mg/day)** | | **Implant D**  **(0.48 mg/day)** | | | |
| --- | --- | --- | --- | --- | --- | --- | --- |
| **Observations** | **Possible**  **Score** | **91 days** | **154 days** | **91 days** | | **119 days** |  |
| Fibrosis | 5 | 2.3+0.5 | 1.6+0.4 |  | 4.2+0.4 | 3.3+1.4 | |
| Inflammatory cells |  |  |  |  |  |  | |
| Polymorphonuclear cells | 5 | 1+0.4 | 1.1+1.0 |  | 2.7+0.7 | 1.4+0.8 | |
| Lymphocyte cells | 5 | 2.3+0.5 | 2.1+1.2 |  | 2.8+0.4 | 3.4+1.0 | |
| Macrophages | 5 | 1.9+0.7 | 1.8+1.3 |  | 2.0 | 1.8+0.5 | |
| Plasma Cells | 5 | 2.0 | 1.5+0.8 |  | 2.0 | 1.8+0.5 | |

Table S8**.** Severity of local toxicity based on histological observations in dogs implanted with TAF implants at either the 3-month midpoint (91 days) or the overall days of completion days for animals assigned to the 6-month timepoint (182 days). Mean (+SD) score from the four dermal sections (cranial, medial, caudal, lateral) submitted for histopathology and rated as the following: Severity: N = Normal (0); 1 = Minimal; 2 = Mild; 3 = Moderate; 4 = Marked; 5 = Severe
